# Foreign Correspondence

**Published:** 1872-11

**Authors:** A. W. Calhoun

**Affiliations:** Vienna, Austria


### FOREIGN CORRESPONDENCE.

**BY A. W. CALHOUN, M. D..**

One of the most dangerous, and yet when timely handled, one of the most yielding manifestations of that loathsome disease Syphilis, is that particular result of the systematic poison which locates itself upon the Iris and is known by the above name. Iritis, when not dependent upon any constitutional cause, in short, when idiopathic, is an enemy, whose approach is well to be dreaded, since its powers of destruction are so familiarly known—but when following upon a general syphilitic affection, and further, when combined with that peculiar characteristic new growth from the substance of the Iris, contributing to the disease the very appropriate additional distinguishing name "Gummosa," then it becomes a matter of the gravest concern to the afflicted individual, whose vision is in the most imminent peril, and to preserve which the most energetic measures must be brought into immediate use.

Iritis gummosa belongs to the symptoms which present themselves early in the secondary stage of syphilis, and is often accompanied, or preceded, by condglomata upon different parts of the body, or by a generel maculous or papulous eruption. These companions, so to speak, of the Iritis, serve as guides to direct us instantly to a minute and positive diagnosis, but were none of these diagnostic aids present and nothing but the intensely inflamed eye itself before us, the picture is so clear and the disease so prominent and characteristic as to bear its own name upon its face and to warn us of the threatening danger.

Upon examining the eye, we find all the appearances of an ordinary Iritis, such as redness and sometimes swelling of the lids, a copious secretion and mostly excessive photophobia, the vessels of the conjunctiva bulbi filled to the utmost, the cornea surrounded by the injected ciliary vessels forming a complete circle, the cornea itself of a cloudy, smoked appearance, and the aqueous humor opaque, often containing floating flakes of exudation. The Iris is discolored, assuming different hues dependent upon its original color, much swollen and upon or near its pupillary margin, a tumor of greater or less size, distorting the already narrowed pupil and jutting forward into the already diminished anterior chamber, towards the posterior surface of the cornea. This tumor decides at once the character of the inflammation, for with very few exceptions, it exists only in Iritis, resulting from syphilis. The seat of this "gumma," is mostly on or near the pupillary edge and consists, as has been fully established by anatomical investigations, of an outgrowth from the parenchyma of the Iris, and is somewhat of that soft or gummy character peculiar to Syphilitic Gummata upon the surface of the body.

Often it is the case that the growth is remarkably rapid, yet in its increase it is never slow. Not unfrequently we find patients who themselves, after a few days inflammation of the eye, discover that in addition to the other appearances the Iris itself is swollen and that on a certain spot something abnormal exists, and are probably the first induced to seek medical aid.

Seldom does the surgeon see his patient before the complete development of the disease, (which requires but a few days) when he finds, as before said, besides the usual accompaniments of an Iritis, situated upon the margin of the pupil and projecting into the anterior chamber, a pale yellow, or yellowish red, more or less broad-based tumor, some portions of which are almost transparent, and passing over and through which are plainly to be seen blood-vessels of different size. This is quite plain to the naked eye, but if we concentrate the light from a lamp or even that from a window by means of a double convex lens, and thus throw it in a focus upon the cornea, moving it from side to side we obtain an exquisite view of the diseased process, and are also readily enabled to distinguish the senechiæ between the anterior capsule of the lens and the posterior surface of the pupillary margin of the Iris, which, to a greater or less extent always take place, even in the first days existence of Iritis gummosa, and which when recent and not extensive, often escape the observer's glance, unless aided by the focal light or by the effects of Atropine.

By ophthalmoscopic examination we discover nothing differing materially from the changes of a common or idiopathic Iritis, viz: a diffused opacity of the cornea, aqueous and vitreous humors, etc., which not seldom, separately or together become so opaque as to prevent the passage of any rays of light through them, and thus, for the time, prohibiting our observations of the interior of the ball.

Under timely and suitable treatment these gummata, as a rule, and the anterior senechiæ (again to be spoken of), oftentimes vanish as rapidly as they appeared, leaving no traces behind them, aside from a discoloration of the Iris on the spot previously occupied by the tumor. But sometimes, when neglected, and occasionally also, but, fortunately, in very rare instances, when under the best management, the gumma continues to increase till the entire anterior chamber is filled and the tumor presses forward and upon the cornea, the Iris taking on suppurative inflammation, causing hypopium, and finally, some of the anterior coats of the eye giving way under pressure and inflammation, a rupture ensues and the whole process ends in Phthisis Bulbi.

Such a sad termination, though one of the rarest, should further stimulate us to an early and active attack upon the disease.

Nor is the affection confined to the adult alone, for there are instances, though seldom, where the sins of the parent are disclosed by the sudden outspringing of Iritis Syphilitica with gumma, in the child. It is a well known and extremely pleasant fact, that Iritis of any kind in children, belong to the most seldom of all diseases, and to further illustrate its rarity, I mention that amongst several thousand patients coming into the Clinics of Profs. Arlt & Jaeger in the last few months, only one such case has presented itself which was that of a little girl eight years of age, who had a truly typical specimen of the Iritis gammosa upon the right eye. That the tumor was the result of a specific inflammation, could not be doubted, since, aside from the appearances upon the Iris, a yellowish red projecting gumma, which alone plainly declared its nature, the fact was undoubtedly established by further symptoms.

Noteworthy is it that the disease is observed only exceptionally amongst females—such instances being comparatively few, either in the different eye departments or in the extensive syphilitic wards or in the wards devoted to skin diseases under the management of Prof. Hebra, where daily an immense number of cases are treated for the multitude of troubles resulting from syphilis in its various stages. The more constant exposure of males to outside hurtful infuences with which females are but seldom brought in contact, may act as a further predisposing cause in the former.

The non-syphilitic form of gumma does now and then come before us, but such is an exception. A few days ago, in the Clinic of Prof. Jaeger, two cases were placed before us, in one of which an excellent example of Iritis gummosa was seen, but from which everything of a specific nature was excluded, not a single trace of syphilis being found or of its ever having existed. In the other was a developed type of syphilitic Iritis, with no symptom whatever of a gumma having once been present or about to form. These two cases were shown to demonstrate the two facts, that Iritis gummosa can occur regardless of syphilis, and that Iritis syphilitica takes place without being complicated by the presence of a gumma.

Although the ordinary syphilitic form of Iritis often occurs upon both eyes at the same time or with but a short interval between its appearance upon the one or the other side, yet fortunately the complication with gummata confines itself mostly to one eye, but few cases being reported in which both at once were similarly affected. This freedom of one eye may be simply accidental, for no valid reasons exist why one syphilitic Iris should be more exempt from such a complication than the other, which may be likewise suffering from the constitutional taint, or why the gummata should not simultaneously spring out from both Irides, each of which may be equally saturated with the constitutional disease.

Why this disease is so dangerous to vision and demands such prompt treatment is perfectly evident to any one who has had many such cases come before him and watched their progress even under the wisest management, or who may have had an opportunity of observing the termination of such or even non-specific Iritis when neglected or mismanaged. We well know how prolific in plastic exudation the Iris is when in an inflamed state, and how prone, through this exudation, the posterior surface, and especially the pupillary margin of the same is, to form adhesions (senechiæ) with the anterior capsule of the lens. And further, we at once see, aside from other evils, that with an abnormal, extremely narrowed pupil and with firm posterior connections preventing its proper dilatation, that the vision, to a certain extent, is already impaired. But were this all, we might soon rid ourselves, as well as the interested individual of any undue concern, but often this is but the forerunner of still greater disasters. In the specific "gummosa" form of the inflammation, the exudation is equally, if not more profuse than in the idiopathic, and is of such a nature as to firmly hold in contact all surfaces where it forms the connecting medium.

A growth of the Iris and anterior capsule, together, even to the slightest extent, can become a source of great trouble to the patient, from a frequent return of a light attack of Iritis. But the tendency is to a circular or ring form growth or senechia, that is, a union between the posterior surface of the Iris in the entire circumference of the pupillary margin, and the adjoining lenticular capsule. Under such circumstances a great fountain of evil is continually present, for in the narrowing of the pupil, during the acute stage of the inflammation, the radiating fibres of the Iris are put upon the stretch, and becoming united to the anterior capsule, the pull upon the Iris substance is continual, and upon the patient contracting slight colds or being exposed to changes of weather, or to other influences which usually would have no dileterious effect whatever upon him locally or constitutionally, just so often is he confined to his room to suffer a fresh onset of his chronic Iritis. Each attack, of course, adds additional fuel to the flames already kindled, implicating often the neighboring parts, such as the Ciliary Bodies, constituting Irido Cyclitis, with all its unpleasant concomitants, most usually leaving the pupil entirely occluded by a false or exudative membrane. Also can the disease extend still further, viz: into the choroidea, giving Irido Choroiditis, which whether going into suppuration or not, ends of necessity in as great, if not total loss of vision, and not unfrequently in panophthalmitis and phthisis bulbi.

As regards the treatment, all might be said in a few words, but although so simple, the importance of attending to certain particulars is so necessary for the sure and rapid success of the especial management required, that I feel disposed not to dismiss the subject till I have gone somewhat into the details of the therapeutics: Of first importance is absolute rest in a darkened apartment. It is scarcely necessary to give a patient advice on this subject, as he has little inclination to expose himself to a light which every moment increases his pain—but his own instinct suggests to him that darkness and quietude give him the ease and comfort so much desired.

In no affection of the eye has the application of atropine been found to render more benefit than in this. In it, the patient, to a great extent, finds his best friend, and the surgeon sees in it his most reliable assistant, without whose aid his hands would be in a state of almost constant cramp. One or two drops of a solution of the atropia sulph. (gr. iij. or jv. to oz. j.) in the lower conjunctival fold will, in a short time, so dilate the pupil and so remove the Iris from the capsule of the lens, that there is no possible way of the slightest senechia taking place even though the exudation may be ever so profuse. This dilatation being once won is of prime importance in the reduction of the disease, and a similar quantity of the solution being daily dropped upon the conjunctiva keeps the pupil in this widened condition and the Iris at perfect rest. Although slight adhesions may have already taken place, the effect of the atropine is often sufficiently strong to relieve the Iris and give it full play. Aside from all this good, atropine is an excellent sedative, relieving, in a great degree, the intense pain so frequently belonging to an Iritis syphilitica with gumma. Even in the very beginning of the disease, where nothing more than a superficial inflammation attracts the patient's or the physician's attention to the eye, and where nothing whatever of a specific nature can be perceived, if it can be discovered that a chancre has several months previously existed, or other symptoms of syphilis are seen upon any part of the body, even then are we authorized to a free use of the atropine. For in the event that the disease progresses to an undoubted Iritis specifica, we have the great advantage of having had the affection already completely in hand before it clearly developed itself, and in case the inflammation ceases without further trouble than its attack upon the superficial structures, we have inflicted, by our caution no further damage than that of giving our patient a few days of quiet, and for a like period disturbing his exact vision through dilatation of the pupil and paralysis of his accommodation, the direct but transitory result of the atropine.

Equally essential for the constitutional treatment, as we have seen atropine to be for the local, is the external use of mercury, viz: by inunction. Where nothing prevents the inunction, mercury is extremely seldom prescribed for internal use. For several years past, in almost all the secondary syphilitic affections collecting themselves in the several eye departments and in the skin and different syphilitic wards, the plan recommended by Professor Sigmund, has been satisfactorily practiced.

For simplicity of explanation we will suppose the treatment to be commenced on the first of the month. On every other day for the first week, the patient is to slowly but thoroughly rub into certain and different parts of the body dr. ss. unguentum hydragiri. Increasing each week dr. ss., and likewise applying it every other day, the individual reaches, on the fourth week the daily use of dr. ij. At the end of this time, requiring a full month, the tumor with the Iritis has usually completely disappeared, and in most cases also the symptoms of the general infection. Should however, any signs of the Iritis or the constitutional trouble still remain, we must continue the daily use of dr. j. ss. or ij. of the ointment, till the disease has vanished or till contra-indications to further use present themselves. As to the propriety of a few days cessation of the treatment and a renewal, must the physician himself be the judge. Contrary to expectation, salivation during the process or as a consequence of these inunctions, is an exception, many cases being at present in the hospital where, as many as fifty or sixty, or even more inunctions have been made without the least unpleasant symptom. This peculiar and surprising exemption from "sore mouth" is greatly due to the continual care exercised, both by the patient and the attendants. From the very beginning, the mouth is several times daily cleansed by a mouth-wash of whatever preparation the physician may prefer, as best acting against the salivating effect of mercury. In the hospitals here the chlorate of potassa is mostly used, and continued so long as the inunctions themselves in the belief that by its simultaneous use it acts in some peculiar manner as a preventive to salivation, and at the same time keeps the patient's mouth free from all effete matter. If, notwithstanding every care and attention ptyalism ensues, the mercury must, of course, be set aside for a time, and renewed, if necessary, when its evil effects have been overcome—to accomplish which every physician has a multitude of remedies at hand. During the course of the treatment one or two baths can be allowed for the comfort of the patient, but to be taken much oftener, are not thought advisable.

The more or less rapid absorption of the tumor can be almost daily observed, usually disappearing completely (under favorable circumstances) in the course of the first week or ten days.

Since writing the above, I have seen a case in the wards of Professor Sigmund, which in progress and result, is a typical illustration of the dangers and occasional termination of this disease, whose picture I have attempted to portray. The patient, a stout, robust man, had a clear case of Iritis syphilitica, but intensified by the addition of an outspoken "gumma" or tumor, such as I have before described, and was being treated by mercurial inunctions, etc. After a few days' treatment the tumor suppurated, depositing its pus in the lower part of the anterior chamber, thus forming hypopium, which, increasing as the tumor grows in extent, also inflamed the cornea, which, in its turn, giving way, a large rupture ensued, permitting also, as the aqueous humor rushed out an extensive prolapse of the Iris. Under a continuation of the inunctions and the local use of atropine and the pressure bandages, this eye is doing as well as could be expected, but in the last day or two, the other eye also has been seized by the same disease, Iritis gummosa, forming an exception to the general rule of its being a "*one-sided affection.*" The result of the disease in this eye can, of course, not yet be seen. The other, the first diseased eye is very probably completely destroyed for all useful purposes.

The object of selecting different portions of the body each day for the rubbing, is quite simple, but the fact was bought by experience. Several successive applications of the ointment upon the same region, with the necessary rubbing and consequently friction, not unfrequently sets up an artificial eczema, which can extend itself over a large part of the body producing fever and other unpleasant symptoms, causing, for a time, the complete suspension of the remedy. To avoid all this, suppose the body, in six or eight divisions, a different disease being taken for each inunction till all the parts have been rubbed, and then return over the same ground, each portion of which has, in the meantime, fully recovered from the irritation produced by the previous inunction.

Internally is given iodide potassium, scr. j. to ij., during the day. All other internal medicament is discarded. Of course, should the mercury act too freely upon the bowels, some preparation of opium is indicated, or if they become constipated, as is sometimes the case, a light laxative must be given.

It cannot be urged that this treatment is suited only for hospital and not for private practice, for were not the urgency of the case sufficient to confine the patient to his room, his own inclinations, his peculiar pain upon exposure to light (photophobia), and the sense of comfort found only in his darkened chamber, lead and retain him there. And while in his private apartments, no interference can well prevent his carrying out the minutiæ of this treatment as fully and effectually as when surrounded by hospital nurses and assistants.

Should, after all, the senechiæ remain unbroken or unloosed, or should there be a frequent return of the Iritis produced either by the senechiæ or from other causes, nothing is left for us but to perform iridectomy, which is often followed by good and permanent results.

The tendency to a return to a specific Iritis, with or without the complication of gumma, (a return of the latter belongs to the greatest infrequency) depends altogether upon the original trouble, a fresh outburst of the syphilis that may still be concealed in his system, at least exposing the individual to the same secondary effects as on the former occasion. We have, however, some consolation in knowing that though the disease return, we still are powerful enough, in a majority of cases, to strip it of its most dangerous and saddest consequences.

VIENNA, Austria, August 20th, 1872.

